# Animal research in the UK: Regulation, implementation, welfare and development of new approach methodologies

**DOI:** 10.1002/ame2.70223

**Published:** 2026-05-12

**Authors:** Ewan St. John Smith, James Bussell, Maggie Gentry, Elliot Lilley, Cathy Merry, Judy MacArthur Clark, William Reynolds

**Affiliations:** ^1^ Department of Pharmacology University of Cambridge Cambridge UK; ^2^ Biomedical Services University of Oxford Oxford UK; ^3^ University Biomedical Services University of Cambridge Cambridge UK; ^4^ National Centre for the Replacement Refinement and Reduction of Animals in Research London UK; ^5^ School of Medicine University of Nottingham Nottingham UK; ^6^ JMC Welfare International London UK; ^7^ Strategic Lead for Science Regulation and Innovation, Home Office London UK

**Keywords:** 3Rs, New approach methodologies, Regulation, Welfare

## Abstract

Use of animals in biomedical research is still considered essential by many in academia, industry and regulatory authorities. Therefore, it is important that legal, governance and welfare procedures are in place to ensure that only necessary procedures using animals are carried out and that this occurs within a framework with animal welfare at its core. Animal research in the United Kingdom is conducted under the Animals (Scientific Procedures) Act 1986 and animal research in the United Kingdom has long been seen as a flag bearer for high quality–high welfare research. An example of the leading role taken in supporting animal welfare in research was establishment of the National Centre for the Replacement, Refinement and Reduction of Animals in Research (NC3Rs) to support reducing the scale and impact of animal research. Here, we provide an overview of governance and licensing procedures of animal research in the United Kingdom, coupled with explanations of how excellent welfare underpins high quality research, and examine the development of new approach methodologies.

## Graphical abstract

Scientific research with animals in the UK is regulated by the Animals (Scientific Procedures) Act 1986 with the National Centre for the Replacement, Refinement and Reduction of Animals in Research providing support for best practice and facilitating development of new approach methodologies. Researchers may only conduct scientific animal research at an institute granted an Establishment License (PEL) under a programme of work authorized by a Project License (PPL) having obtained their own Personal License (PIL); all licenses are awarded by the Home Office. Provision of excellent animal welfare results in high quality and reproducible research, progress with new approach methodologies aiming to reduce and replace use of animals.

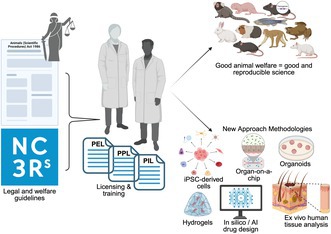

## 1 HISTORY

Regulation of animal research in the United Kingdom (UK) emerged from a confluence of scientific advancement and societal concern that began in the 19th century. The Cruelty to Animals Act 1876, responding to public campaigns, established the world's first comprehensive legal framework for laboratory animal protection, requiring licenses for experiments and introducing the principle that animal suffering must be justified by scientific necessity.^1^ Although France introduced the Grammont Law earlier, in 1850, this only applied to public abuse of animals and did not directly regulate scientific experimentation with animals, the United Kingdom having also introduced the Cruel Treatment of Cattle Act 1822, which is often considered the first piece of animal welfare legislation globally.^2^

This pioneering approach reflected the UK's position as being at the forefront of modern experimental science and home to the world's first animal welfare movements. The 1876 Act's core philosophy – that scientific progress and animal protection must coexist – became a defining characteristic of UK policy and influenced regulatory thinking across Europe and beyond.

The Animals (Scientific Procedures) Act 1986 (ASPA)^3^ transformed this Victorian framework into modern legislation, introducing the “three Rs” principle (Replacement, Reduction, Refinement) developed by Russell and Burch in 1959.^4^ ASPA established comprehensive project evaluation, mandatory cost–benefit analysis, and independent ethical review, innovations that positioned the UK as a global leader in humane research regulation. Although beyond the remit of this review, it is important to note that in addition to legislation regarding use of animals in scientific research, the UK has also been at the forefront of developing guidance to support welfare of livestock animals. The ‘Brambell Report’ of 1965 stated that animals should, “have sufficient freedom of movement to be able without difficulty, to turn round, groom itself, get up, lie down and stretch its limbs”.^5^ In response to the report, the Farm Animal Welfare Advisory Committee was established, now called the Farm Animal Welfare Committee, which codified the Five Freedoms: Freedom from hunger and thirst, Freedom from discomfort, Freedom from pain, injury or disease, Freedom to express normal behavior, and Freedom from fear and distress^6^; guidance incorporated by institutes worldwide, including the World Organization for Animal Health.^7^ In more recent years, the Five Freedoms have been built upon further in terms of the Five Domains Model for animal welfare assessment, which encompasses: Nutrition, Physical Environment, Health, Behavioral Interactions and Mental State.^8^

The UK's expertise became internationally significant when the European Union adopted Directive 2010/63/EU, heavily influenced by ASPA's framework.^9^ UK scientists and regulators contributed substantially to the Directive's development, sharing decades of practical experience in implementing welfare‐based regulation without compromising scientific excellence. This regulatory evolution reflects broader social changes in the UK and Europe, where public expectations of transparency and animal welfare have continuously risen. The establishment of the UK National Centre for the 3Rs (NC3Rs, see below) demonstrated governmental commitment to advancing alternatives while maintaining scientific rigor, a balance that resonates with approaches being developed globally.

The UK approach has proven valuable in broader international partnerships, with productive collaborations over the past decade including work with China's scientific community.^10^ These partnerships demonstrate how established regulatory frameworks can contribute to global advancement through mutual learning and shared expertise, benefiting both scientific progress and animal welfare across different cultural contexts.

## 2 NATIONAL CENTRE FOR THE REPLACEMENT, REFINEMENT AND REDUCTION OF ANIMALS IN RESEARCH

The National Centre for the 3Rs^11^ (NC3Rs) was established in 2004 in response to a UK Government recommendation to strengthen the application of the 3Rs in science. It pioneers better science by working collaboratively with researchers, industry, regulators and policymakers in the UK and internationally to develop, validate and implement approaches that replace, reduce and refine the use of animals in research and testing.

Over more than 20 years, the NC3Rs has transformed the level of engagement, activity, acceptance and use of 3Rs approaches, delivering scientific, ethical and economic benefits. A defining feature of its success is its ability to act as an independent, trusted “honest broker”, bringing together diverse international stakeholders to overcome scientific, regulatory and practical barriers to change.

A recent example of an NC3Rs project was work undertaken to review World Health Organization (WHO) guidance for the quality control of vaccines and biological therapeutics.^12^ Up to 10 million animals a year are used worldwide in these tests,^13^ which are expensive, can cause significant pain and distress to the animals, and can be highly variable, causing lengthy delays to the release of key medicines.^14^ With funding from The Gates Foundation, the NC3Rs convened an international group of experts spanning biological manufacturers, regulators and other key stakeholders to support a review of WHO guidelines and written standards, and recommended revised language to focus on replacement approaches where they are validated, and ensure refinements are included where they are not. In direct response to NC3Rs recommendations the WHO published a new guideline in 2025 that strongly encourages manufacturers and regulators worldwide to replace or remove animal‐based quality control methods whenever scientifically justified.^15^ This represents a significant step towards global harmonization, with the potential to reduce animal use on a large scale while improving scientific robustness, efficiency and animal welfare.

More broadly, the NC3Rs delivers its mission through targeted research funding, innovation programmes and expert‐led policy engagement. Activities are focused on four areas: improving the experimental design, analysis and reporting of in vivo and in vitro research; working with funders and universities; embedding the 3Rs in pharmaceutical and chemical safety regulations; and championing animal welfare across the life sciences. NC3Rs is the largest funder of 3Rs research in the UK, committing £7–8 M per annum for research^16^ and innovation,^17^ and building capacity by supporting early career researchers to focus on the 3Rs.

NC3Rs' approach demonstrates how coordinated international collaboration, grounded in scientific credibility and regulatory trust, can accelerate the global adoption of non‐animal technologies and deliver lasting improvements for both science and animal welfare.

## 3 LICENSES AND TRAINING

To conduct animal research under ASPA, three licenses are required from the Home Office:

1. An Establishment License (PEL), which authorizes an institution to maintain and use protected animals for scientific procedures, placing legal responsibility on the institution for facilities, governance, staff, veterinary care and welfare to support research.
2. A Project License (PPL), which authorizes a programme of work, justifying scientific objectives, species/numbers to be used, procedures to be conducted, and application of the 3Rs. Obtaining a PPL often takes up to 12 months and the maximum period covered is 5 years; PPLs cover a complete body of work and are non‐renewable, thus, a new PPL is applied for as required.
3. A Personal License (PIL), which authorizes an individual to conduct regulated procedures on defined species after receiving training and obtaining competence in each procedure. Training and examination (theory and practical) are overseen by the Home Office.

To obtain a PPL, an investigator writes a first draft with institutional animal welfare experts before submission to the institution's Animal Welfare Ethical Review Body (AWERB, comprising internal and external members). Feedback must be acted upon before submission to the Home Office via the Animals in Science Procedures e‐Licensing (ASPeL) website. A Home Office Inspector (HOI, usually a veterinary surgeon with research experience) has 40 working days to review, providing feedback that needs to be addressed before an application is authorized or rejected, this process can involve multiple rounds of review.

Once granted, a PPL can be amended by following the same AWERB, ASPeL and HOI pipeline: it is a living document. Under ASPA, certain PPLs are required to undergo a Retrospective Assessment (RA) at the point of expiry. This requirement is set at the point the license is granted, and the RA is submitted to the HO and AWERB. RAs are mandatory for PPLs that involve: non‐human primates; procedures classed as severe; use of specially protected species; endangered species and all those for education and training purposes. Those PPLs not requiring an RA undergo Retrospective Review, which, like the RA process, specifically seeks to evaluate the advances achieved by the research, the harm experienced by the animals and how this compares with the conditions of the authorized license, the review process overseen by AWERB.

The robustness of the PPL awarding and amending process cannot be questioned, but the administrative burden on the person applying for the PPL, as well as those involved in the assessment process should be acknowledged, as well as the time the process can take before a investigator is able to undertake a body of work. There are potential options for accelerating the process, such as the establishment of more HO‐approved Protocols; these would sit alongside those already in place relating to breeding and maintaining genetically altered animals. HO‐approved Protocols provide greater reproducibility in terms of what is authorized and conducted across institutions, rather than having assessors (either in AWERB or the HO) having to make fine judgments when assessing essentially the same Protocol when submitted by different investigators at different institutes. The opportunity to standardize yet retain the ability for amendment to an HO‐authorized Protocol thus maintains standards of protections to animals, provides investigator flexibility and minimizes the administrative burden on those writing and assessing Protocols.

PPL compliance breaches – for example, an animal dies unexpectedly whilst undergoing a procedure – are reported back to the local institution and Home Office via a Standard Condition 18 form. The form includes fields for discussing how to avoid repetition and involves input from the institute's Named Care and Welfare Officer (NACWO) and Named Veterinary Surgeon (NVS). This process is important for maintaining transparent regulatory oversight of animal welfare in research.

**FIGURE 1:**
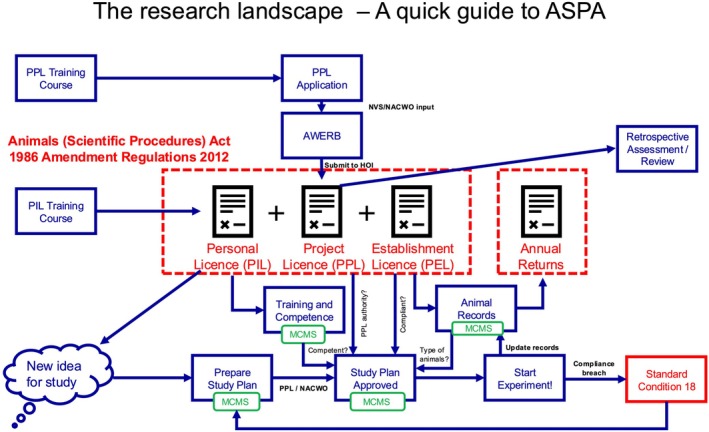
Summary of UK framework for conducting animal research showing the Home Office licensing and monitoring systems (red) alongside the training and steps taken by the researcher to obtain training and conduct research (blue), and how MCMS can be used to support the process (green); with assistance from Stuart Pierson.

Although a PPL authorizes conducting of procedures, most institutions require Study Plans (or something similar) to be submitted by a PIL holder for each experiment, and these require approval by the PPL holder and NACWO. Many institutions use the Mouse Colony Management System (MCMS), an online portal for Study Plan submission, review and approval. Study Plans provide details of what will take place and when: animals and methods to be used with anticipated adverse effects and humane endpoints, substances to be used with appropriate safety information, day‐by‐day procedural breakdown, and an ability to cross check the procedures against the trained competencies of those undertaking the study. Once approved, labels are added to cages to aid technicians providing daily care. PIL holders must be appropriately trained by a competent researcher and assessed by a designated trainer with regular reviews to ensure that competency is maintained; training records are maintained in MCMS. Annual returns of animal usage and severity levels are reported back to the Home Office and MCMS can be used to generate the required data. Figure 1 provides a summary of these processes.

## 4 EVOLVING WELFARE TO FACILITATE RESEARCH

With a focus on the mouse as a model organism, we have seen a huge progression in welfare in an everchanging scientific landscape. The turn of the 20th century saw creation of some of the first inbred mouse strains such as the C57Bl/6^18^ in an uncontrolled environment. These strains have been fundamental to controlling the genetics and understanding how alteration in a background genome enables understanding the function of individual genes. Innovations in genetic modification through naturally occurring mutations, chemical mutation, random insertion, targeted knockouts and on to modern endonuclease mutagenesis combined with genomic sequencing have permitted further dissection of these functions.

Such mutations have also driven the way animals are housed, monitored and provided for, leading to the evolution of greater regulation of the care of the modern laboratory animal. Law and legislation have helped drive consistency and development of better housing by laying out the conditions that should be applied to a wide range of species. During the 100 years since the first genetically identical mice were developed, control over all aspects has been achieved. This can be seen in caging systems moving from open caging using materials that harbor pathogenic organisms through to sterilisable caging which forms a microenvironment with controlled temperature, humidity, bedding, defined diets and high‐quality water.

The modern research animal is a highly controlled organism with a well characterized background. The knowledge of expected phenotypes and alteration to the genome allows experimental consistency and the monitoring of their welfare. Indeed, good welfare should be at the centre of all animal research: high standards of animal welfare and science go hand‐in‐hand. Knowing and understanding the signs of wellbeing in the animals is paramount to obtaining robust results and reducing variables in the science. It has been proposed that there are three ways in which animal wellbeing can be viewed: health and biological functioning, the degree of natural living, and the affective states.^19^, ^20^ How the wellbeing of laboratory animals is supported and assessed cuts across these viewpoints; for example, advances in home cage enrichment technologies that improve the degree of natural living have improved the environment for laboratory animals, resulting in decreased morbidity.^21^, ^22^

For example, a group studying anxiety and depression in marmosets must ensure that the marmosets they work with are free of anxiety in their home surroundings. Cage design, considering height and depth of the cage to enable movement away from humans, should they wish, is critical. Furthermore, caging that enables social interaction between neighbors and reducing fright by permitting the marmosets to see who is entering the room both yield beneficial welfare outcomes. Other considerations include the reduction of cold and noisy materials through use of wood and rope. Providing fresh, natural foods, such as oranges to peel, and scattering live insects in the pen all enable natural hunting and foraging behaviors.

The scientific value of high standards of animal welfare has also been demonstrated in mice with experimental autoimmune encephalitis (EAE), a disease leading to partial and/or complete paralysis of the hindlimbs. Welfare must be of a high standard to ensure survival of the mice so that data can be collected. Mice with EAE have reduced ability to reach the food hopper and waterspout. When clinical symptoms of EAE become evident, replacing the aspen bedding with a cloth surgical pad improves the mobility of mice, through increased traction on the pad, as well as reducing occurrence of sores. Mice with EAE lose sensation in their tail and limbs, which can result in autophagia. However, by replacing normal nesting material with a cardboard house and small pieces of torn cardboard, an outlet is provided for the mice to bite and chew the cardboard instead of their limbs. Other welfare support includes using cardboard dishes with the side removed to provide nutrient gels, providing pellets on the floor, and attaching heat pads to the bottom of the cage as the reduced mobility of the mice means they cannot control their body temperature as well.

Animal welfare and science are not two separate entities but one combined method of improving scientific quality and reproducibility of our research.

## 5 NEW APPROACH METHODOLOGIES: PROGRESS AND CHALLENGES

New Approach Methodologies (NAMs) are a suite of innovative experimental and computational tools that currently mostly augment, but may soon replace, traditional animal‐based testing, where it is safe and sensible to do so. The UK's approach to NAMs is for them to be delivered through a coordinated, cross‐government strategy that combines regulatory oversight under ASPA with targeted investment, scientific leadership and policy levers to accelerate the development, validation and regulatory acceptance of NAMs while maintaining confidence in safety and efficacy assessment.^23^ At their most effective, NAMs offer enhanced human biological relevance and mechanistic insight at lower cost and with improved ability to reflect genetic, age, and sex variance in the population. Aligned with changes to legislation around the use of NAMs in toxicity screening, cosmetics testing and mechanistic or efficacy studies, there has been a rapid expansion in the range of approaches and the numbers of labs developing them. Central to this are microphysiological systems (MPS) such as organs‐on‐chips (OoCs), advanced computational models including artificial intelligence (AI), and engineered three‐dimensional (3D) biomaterials. These aim, independently or together, to provide improved human‐realistic environments that can support chemical and drug evaluation, at scale.

There have been some excellent reviews on this topic, including a commentary on MPS standardization,^24^ the application of advanced mathematical and computational modeling to generate model informed drug discovery^25^ and 3D models of disease, with innovations in cancer modeling,^26^ and highly challenging diseases such as neuropsychiatric disorders.^27^

## 6 OoC SYSTEMS

OoC technologies are microfluidic devices that recreate key aspects of human tissue architecture, mechanical forces, and biochemical gradients on a miniature platform, enabling some level of functional mimicry of human organs at the cellular level. These systems integrate human cells into precisely controlled microenvironments that permit real‐time assessment of physiological processes relevant to toxicology, pharmacokinetics, and disease modeling. OoCs have clear advantages over static 2D culture, enabling a more tissue‐realistic environment and the inclusion of multiple cell types. Improved recapitulation of essential phenotypic and functional tissue features strengthens translational relevance for drug screening and personalized response prediction.

Recent advances have extended OoC applications across multiple organs, including lung, heart, gut, and vasculature, and prompted development of multi‐organ configurations (or body‐on‐chip) to simulate systemic interactions and inter‐organ toxicity^28^; for example, creation of bi‐directional gut‐brain axis models, incorporating a blood–brain‐barrier component, applied to the relationship between gut bacteria and neuroinflammation.^29^ Whilst these complex systems are impressive, often (as in this case) they still make extensive use of animal‐derived materials including matrix products and growth factors, although efforts are being made to reduce such reliance.

## 7 AI AND PREDICTIVE COMPUTATIONAL APPROACHES

Artificial intelligence and machine learning have emerged as powerful analytical tools within NAMs, with the aim of extracting meaningful patterns from large, multidimensional datasets and informing predictive models of biological responses. AI algorithms are increasingly applied to toxicology and drug evaluation to predict hazards, optimize experimental design, and integrate heterogeneous data streams from high‐throughput assays and MPSs. The potential benefits of including AI have recently been discussed, including increased efficiency and accuracy, as well as better integration of diverse datasets. However, related potential drawbacks included issues with data quality, model interpretability and regulatory acceptance.^30^

An application where AI is already proving useful is in the validation of NAMs. Suggestions for how this can best be achieved include the smart selection of reference chemicals, simulating validation studies and mechanistic validation. However, the success of AI approaches in this space relies on strong interdisciplinary collaborations or the training of a new generation of researchers, and legislators with skills in both biological and AI models,^30^ as well as the environmental impacts associated with AI usage and large‐scale data storage.

## 8 HYDROGELS AND SCAFFOLDS AS 3D CULTURE MATERIALS

The extracellular matrix (ECM) that surrounds cells and controls and is integral to the individual properties of different tissues is essentially a mesh of fibrous and gel‐like components. It is not surprising therefore that, in creating synthetic models of tissues, researchers often turn to biomaterials that mimic these properties. Broadly, approaches can be split into those that make use of animal‐ or human‐derived components (e.g. Matrigel, extracted from mice with an ECM‐producing tumor) and those created from synthetic components. Both approaches have benefits and limitations, but there is now a strong push to move away from complex, poorly reproducible animal‐derived materials. Synthetic scaffolds and hydrogels can often be engineered to carry specific motifs that enable cell‐binding, or to have mechanical properties that, for example, drive differentiation of stem cells to particular lineages.

Cancer research and a move towards personalized disease modeling using 3D models is particularly well advanced.^31^ At least in part, this is due to the well‐established role of the ECM in controlling cancer behavior and the need to create models that better recapitulate the tumor microenvironment.

## 9 INTEGRATION TOWARDS PERSONALIZED MEDICINE

Much of the excitement around NAMs is the integration of the approaches described above with patient‐relevant material (e.g. tumor biopsies, patient specific induced pluripotent stem cells and organoids etc.) to create MPSs enabling the direct study of inter‐individual variability in drug responses and toxicity profiles.^31^ By linking in AI‐driven analysis of the data, perhaps correlating with patient medical histories, the approaches support stratified positions that can guide improved clinical decision‐making, customized treatment strategies and improved treatments for genetically diverse populations.

## 10 LIMITATIONS OF NAMs

For all the exciting progress being made with NAMs, there are limitations. Firstly, whole organisms are complex with many different organ systems interacting with each other, which is still impossible to fully replicate in silico or in vitro, and thus there is currently still a need for examining in vivo function or efficacy. For example, a recent paper demonstrated the ability to grow esophageal grafts from mesenchymal cells derived from recipient minipigs, but implantation in vivo was required to measure functional integration, with 63% of animals surviving to the 6‐month endpoint.^32^ Moreover, many NAMs can only examine specific questions, such as computational molecular modeling being able to assess how a compound may interact with different receptors, but what the physiological effects are of such receptor interactions, in different tissue types that interact with each other, currently requires more complex, in vivo examination. Regulatory acceptance is a further hurdle to be overcome, but as NAMs further develop then these hurdles may prove easier to jump, as indicated by the WHO's guideline discussed above. Overall, in the short‐term, improved integration of NAMs with well‐regulated and well‐conducted in vivo research has great potential to reduce total animal usage to make sure that fewer, better justified and better designed experiments are undertaken.

## 11 SUMMARY

In conclusion, appropriate regulation and governance, alongside high‐quality welfare, supports excellence and reproducibility in scientific research with animals, with institutions like the NC3Rs facilitating NAM development in an effort to reduce and replace use of animals in research.

## AUTHOR CONTRIBUTIONS

**Ewan St. John Smith:** Conceptualization; writing – original draft; writing – review and editing. **James Bussell:** Writing – original draft; writing – review and editing. **Maggie Gentry:** Writing – original draft; writing – review and editing. **Elliot Lilley:** Writing – original draft; writing – review and editing. **Cathy Merry:** Writing – original draft; writing – review and editing. **Judy MacArthur Clark:** Writing – original draft; writing – review and editing. **William Reynolds:** Writing – original draft; writing – review and editing.

## FUNDING INFORMATION

No funding directly supported the writing of this article.

## CONFLICT OF INTEREST STATEMENT

Authors declare no conflicts of interest.

## ETHICS STATEMENT

None.

## Data Availability

N/A.
